# “A Somali girl is Muslim and does not have premarital sex. Is vaccination really necessary?” A qualitative study into the perceptions of Somali women in the Netherlands about the prevention of cervical cancer

**DOI:** 10.1186/s12939-015-0198-3

**Published:** 2015-08-21

**Authors:** Jihan Salad, Petra Verdonk, Fijgje de Boer, Tineke A. Abma

**Affiliations:** Department of Medical Humanities, VU University Medical Centre, EMGO+ Institute of Health and Care Research, Amsterdam, The Netherlands; United Nations Complex, United Nations Avenue, Block P, Level 2, Somali, Kenya; Department of Medical Humanities, VU University Medical Centre, EMGO+ Institute of Health and Care Research, School of Medical Sciences, Amsterdam, The Netherlands

## Abstract

**Introduction:**

Participation in Human Papillomavirus (HPV) vaccination and Papanicolaou Screening (Pap smears) is low among ethnic minorities in the Netherlands and hardly any information is available about the cervical cancer prevention methods of Somali women living in the diaspora. This qualitative study, based on the Health Belief Model (HBM) and an intersectionality-based framework, explores the perceptions of Somali women living in the Netherlands regarding measures to prevent cervical cancer.

**Methods:**

Semi-structured interviews have been conducted with young Somali women aged 17–21 years (*n* = 14) and Somali mothers aged 30–46 years (*n* = 6). Two natural group discussions have been conducted with 12 and 14 Somali mothers aged 23–66 years. The collected data has been analyzed thematically for content.

**Results:**

In this study, we have identified perceived barriers to the use of preventive measures across three major themes: (1) Somali women and preventive healthcare; (2) Language, knowledge, and negotiating decisions; and (3) Sexual standards, culture, and religion. Many issues have been identified across these themes, e.g., distrust of the Dutch health care system or being embarrassed to get Pap smears due to Female Genital Mutilation (FGM) and having a Dutch, male practitioner; or a perceived low susceptibility to HPV and cancer because of the religious norms that prohibit sex before marriage.

**Conclusions:**

Current measures in the Netherlands to prevent women from developing cervical cancer hardly reach Somali women because these women perceive these kinds of preventative measures as not personally relevant. Dutch education strategies about cervical cancer deviate from ways of exchanging information within the Somali community.

Teachers can provide culturally sensitive information to young Somali women in schools. For Somali mothers, oral education (e.g., poetry or theater) about the Dutch health care system and men’s roles in HPV transmission may be useful.

An intersectional approach, grounded in the HBM, is recommended to promote equal access to preventive health care for Somali women.

## Background

**Table:** 

Soomaali buraanbur	Somali poem^1^
“Ka hortag kankarada afka ilma-galeenka”	“Prevent Cervical Cancer”
Ho’hooy ho’hooy haween dumar	Oh oh [you] women
Horta maxaad isku hiiftaan?	Why don’t you take care of each other?
Dantiina u hagrataan?	Why don’t you invest in yourself?
Hooyiga caafimaadka intaad tagtaan	Why don’t you go to the health center and,
hoos ma isku baartaan?	examine yourself down under [cervix]?
Heerkiina caafimaad	Why don’t you check-up your health [in general]
iyo akhbaar hubsan ma doontaan?	and seek reliable information?
Cuduro hortag lehbaa milay iyo malyuumaad	Preventable diseases have affected millions of people
hilafa u qaadoo badhkood iilka loo dhigay	and many of them died without knowledge [of the prevention of diseases]
Heedhe shalay shaley aheeyd	Yesterday was yesterday
Haatan dunidu waa hormar	The world has progressed
heer sareey mareeysaa	and is now advanced
Hadeeysan taadii Alle gelin	If it isn’t God’s plan [to take your life away]
Ka hortag dhimashaa la helay	Prevention of death [from diseases] is found
Difaac cudur baa la helay	Protection against diseases is found
Baarid caafimaad oo hufan baa la lehay	Good medical check-up practices are found
Adoon howl yareeysan, isku baar hagar la’aan	Take it [disease] seriously and do a [medical] check-up
Oo ka hortag kaankarada afka-ilma galeenka	To prevent cervical cancer
iyo dhamaan cudurada haweenka ku habsaday	and all other diseases that affect women
Yasmin Ali, Somali poetess	

Since 2009, prevention of cervical cancer in the Netherlands consists of two measures: first, the Papanicolaou Screening (Pap smear), for which all women aged 30 to 60 years receive an invitation every 5 years, is free of charge [1]. The second measure - the Human Papillomavirus (HPV) vaccination - was introduced in 2009, after the bivalent vaccine (Cervarix) targeting HPV 16 and 18 became available [2]. Since there is a risk of contracting HPV from the first time of intercourse [3], the HPV vaccination program, which is managed by the Centre for Infectious Disease Control (CIb) and is only free of charge for 12-year old girls [2], ensures that most girls can be vaccinated before they become sexually active [4].

Although the goal for HPV vaccination coverage was set at 70 % [4], less than half of invited girls born between 1993–1996 (45 %) showed up to receive all three injections during the campaign of 2009, when the vaccination program started [2]. Also in 2013, the vaccination coverage remained low: slightly more than half of the girls born in 1997 (56 %) and 1998 (58 %) received the three injections [5].

Participation in HPV vaccination and Pap smears is particularly low among ethnic minorities in the Netherlands [1, 4], however, the morbidity of cervical cancer in the Netherlands is also low among ethnic minorities. According to a Dutch study, statistically significant lower numbers of cervical cancer related deaths were identified among ethnic minority women when compared to native Dutch women: 35 compared to 2922 cases, respectively [6].

Some studies suggest this low risk may be due to having circumcised partners [7], among other reasons, male circumcision being a common practice in Islamic communities [8].

The Somali community in the Netherlands, which is predominantly an Islamic community [9], is under studied in health research in general. There is very little published research on the participation of Somali women in the Netherlands in measures to prevent cervical cancer. Health research in the Netherlands about Somali women particularly focuses on (the prevention of) FGM [10]. However, limited research has explored how FGM may form a barrier in the prevention of cervical cancer among Somali women (Table 1).

**Table 1:** Somali people in the Netherlands

The Somali community in the Netherlands is comprised of over 34,000 people [42]. The first group of refugees, particularly higher educated individuals, fled to the Netherlands after the Somali civil war broke out in 1990 [9, 43]. The second group of refugees, mainly lower educated individuals, fled to the Netherlands after 2006 [9, 44].
In Somalia, female genital mutilation (FGM) is common practice [45], next to male circumcision [46]. Somali women’s lives in the diaspora are not only influenced by their traumatic experiences because of war and FGM, but also by their migration history, which has led to social and legal barriers such as a devalued refugee identity, unemployment, social isolation, thwarted aspirations, and changing gender roles. These factors have been linked to a prevalence of mental and physical problems [10, 47, 48].
In the Netherlands, a variety of Somali foundations and associations provide support for social integration [44]. Most information is communicated orally because of the prevalent oral culture within the Somali community [41].

In Somalia, death due to cancer is relatively low (4 %) [11]. However, cervical cancer is the second most common cancer among women in Somalia. It is estimated that nearly 3 million Somali women aged 15 years and older are at risk of developing cervical cancer. Each year, 967 women are diagnosed with cervical cancer and 546 die from this condition [12]. The high incidence of cervical cancer deaths may be explained by the lack of preventive cervical cancer care, as HPV vaccinations and Pap smears are not implemented in Somalia [13, 14].

In the United Kingdom, embarrassment due to Female Genital Mutilation (FGM) is a barrier for getting Pap smears among Somali women [13]. In the United States (US), FGM is associated with a concern that Pap smears would increase pain among Somali girls and women [14]. Another study in the US has found that Somali girls accept HPV vaccination, but are less likely to complete the HPV vaccination series in comparison with white non-Hispanic girls [15]. Moreover, Somali women in the US are less likely to adhere to cervical cancer screening procedures than non-Somali women in the US [16]. Hence, there is a need to deepen our understanding of the perceptions of Somali women in the diaspora regarding the prevention of cervical cancer.

In this paper, we refer to “sex” as the biological characteristics of a person, such as genetics, hormones, and reproductive organs, and to “gender” as the socio-cultural aspects attached to being male or female in a given context, hence, to the social and relational processes in various contexts [17]. In order to understand how social identities such as gender, culture, and religion relate to each other and influence the participation of Somali women in measures to prevent cervical cancer, we use the concept of intersectionality as part of our theoretical framework [18]. Intersectionality, which has its roots in social justice within societies, aims to address how social identities like gender and cultural background affect life, and refers to how they constitute interactive relationships that influence each other. It directs attention to how a single focus on gender, culture, ethnicity, age, or religion, on its own, is insufficient for studying the relational nature of social forces and localities that shape lived experiences. Acknowledging the role of one factor may be important, but it should not be disconnected from other categories because this would limit the contextualization of the analysis [19, 20].

**Fig. 1:**
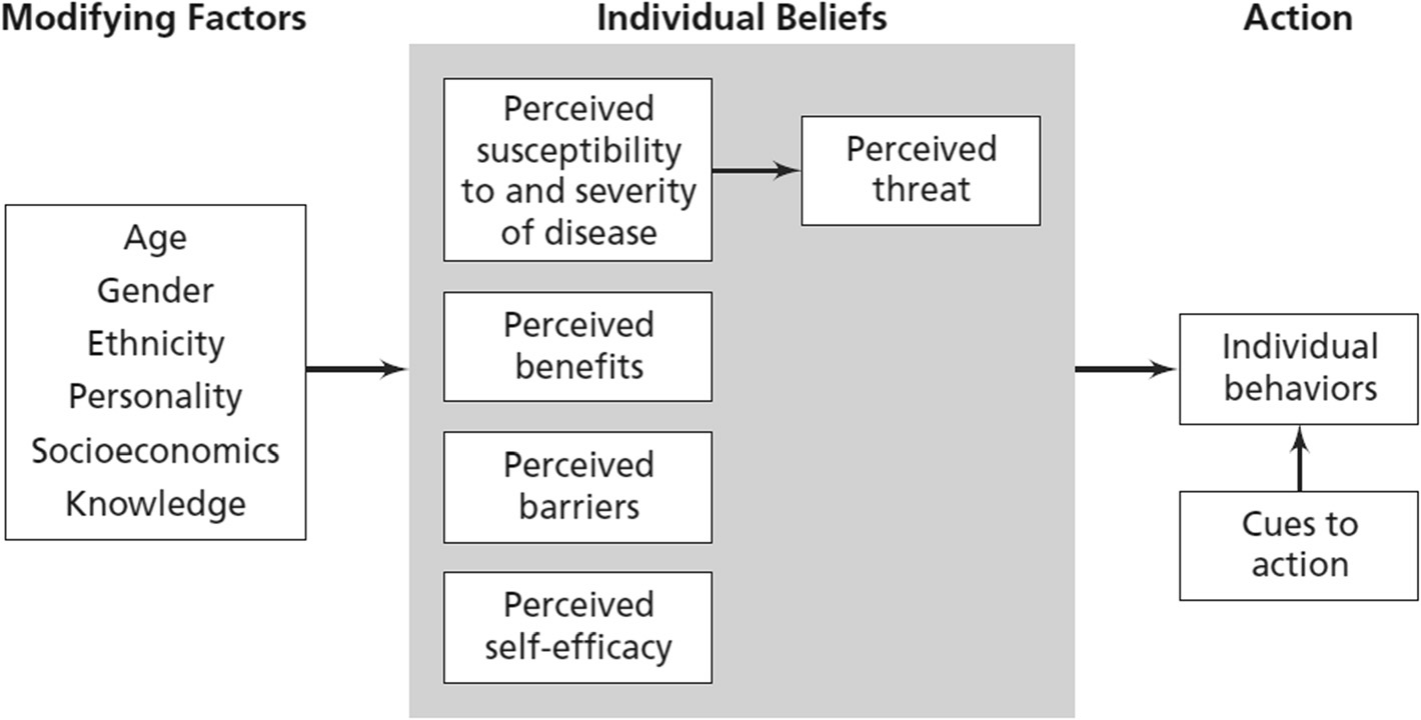
The health belief model [21]

A second theoretical approach that is used in this study is the Health Belief Model (HBM). According to the HBM, health-related action depends on perceived susceptibility, perceived severity of the disease, perceived benefits of actions or measures, perceived barriers to action, cues to action, self-efficacy, and modifying factors [21] (Fig. 1).

Health beliefs, however, are informed by people’s social identities. Intersectionality is therefore integrated in the HBM in this study, which aims to explore the perceptions of Somali women living in the Netherlands regarding measures to prevent cervical cancer.

## Methods

A qualitative approach has been used in this study to gain insight into the personal experiences and views of Somali women concerning HPV, and to discuss sensitive topics such as sex [22]. Interviews and natural group discussions have been used as methods to collect data.

### Recruitment methods and site selection

The executing researcher (JS), who is a young female of Somali origin and studied Health Sciences in Amsterdam, approached Somali organizations and Community Health Services (CHSs) through gatekeepers who had access to the Somali community. Participants were recruited from a wide range of settings, including community gatherings, CHSs, the university, and Facebook. The recruitment was mostly done by JS and via members of the Somali organizations and CHS professionals.

The young Somali women, between 18 and 21 years old, were recruited because they have been a part of the target group for the first (catch-up) HPV vaccination program, and they will be invited for Pap smears when they turn 30. The mothers who were over the age of 30 were recruited in this study because they had either been invited to receive Pap smears or because they had to decide whether their 12 year old daughters would receive the HPV vaccination.

The inclusion criteria has been comprised of being female and of Somali origin, living in the Netherlands, aged between 18 and 65, and having a migration date from the first or second wave of migration (see Table 1). Daughters whose mothers have participated in the study were excluded and vice versa because sexuality issues and health issues are sensitive to discuss in the Somali community, and it would have limited building rapport between the interviewees and the interviewer. Furthermore, convenience sampling has been used at community gatherings of the Somali women.

Purposive sampling has been used to collect information from participants with a varied background. Snowball sampling has been applied and possible participants have been checked to see whether they fulfill the inclusion criteria for this study.

### Characteristics of the study population

In this study, 14 young Somali women aged 18–21 years and six Somali mothers aged 30–46 years were individually interviewed (Table 2).

**Table 2:** Characteristics of Somali women who have participated in individual interviews

N	Young Somali woman/Somali mother	Country of birth	Age	Age at migration	Education level	Intention to/do not accept/reject/Human Papillomavirus (HPV) vaccination^a^	Intention to/do not accept/reject/Papanicolaou Screening (Pap smears)
1	Somali mother	Somalia	46	31	Middle-level applied education	Accept	Accept
2	Somali mother	Somalia	43	30	Middle-level applied education	Reject	Accept
3	Somali mother	Somalia	30	19	No education	Reject	Reject, but would accept now
4	Young Somali woman	Somalia	18	Under five	University of applied science	Reject	Not applicable
5	Young Somali woman	Somalia	21	Under one	University of applied science	Not invited, but would accept	Not applicable
6	Young Somali woman	The Netherlands	18	Not applicable	University of applied science	Reject	Not applicable
7	Young Somali woman	The Netherlands	21	Not applicable	University of applied science	Not invited, but would accept now (and reject in the past)	Not applicable
8	Somali mother	Somalia	32	28	No education	Accept. However, daughters are below the age of 12	Reject, but would accept now
9	Somali mother	Somalia	33	31	No education	Accept. However, daughters are below the age of 12	Reject, because pregnant. Participant would accept now
10	Young Somali woman	The Netherlands	21	Not applicable	University of applied science	Not invited. Unknown whether participant would accept or reject	Not applicable
11	Young Somali woman	The Netherlands	21	Not applicable	University	Not invited. Unknown whether participant would accept or reject	Not applicable
12	Young Somali woman	Egypt	20	Not applicable	Middle-level applied education	Not invited, but would reject	Not applicable
13	Young Somali woman	The Netherlands	18	Not applicable	University of applied science	Rejected by mother. Participant would accept the vaccine now	Not applicable, but would reject Pap smears
14	Young Somali woman	Somalia	21	Four	University	Participant lost the invitation letter. Participant would reject the vaccine now	Not applicable
15	Young Somali woman	The Netherlands	18	Not applicable	University of applied science	Accept. However, participant received 2 injections. According to her, she completed the HPV vaccination series	Not applicable
16	Young Somali woman	Saudi Arabia	20	Not applicable	Middle-level applied education	Participant does not know whether she received the invitation letter. Participant would reject the vaccine	Not applicable
17	Young Somali woman	The Netherlands	19	Not applicable	Middle-level applied education	Reject	Not applicable
18	Young Somali woman	Syria	19	Not applicable	Middle-level applied education	Rejected by mother, but would accept the vaccine now and in the past	Not applicable
19	Young Somali woman	The Netherlands	19	Not applicable	University	Reject	Not applicable
20	Somali mother	Somalia	35	30	No education	Does not remember an invitation, but would accept the vaccine	Did not receive an invitation, but would accept

^a^The Somali mothers were questioned about their daughters’ HPV testing status, while the young Somali women were asked about their own HPV testing status

All of the mothers in the study were born in Somalia. Three mothers were part of the first migration wave (after 1990) and three of the second wave (after 2006). Two mothers from the first migration wave obtained education in Somalia and additional education in the Netherlands. Two mothers had received a Pap smear earlier, while three declined the invitation. One mother did not receive an invitation.

Nearly half of the Somali girls were born in the Netherlands (8 out of 14), the other girls were younger than the age of 5 at the time of migration to the Netherlands. Most of the young Somali women are highly educated (10 out of 14). All the girls had received childhood vaccinations, while only one out of the eight invited girls received the HPV vaccination. Five girls did not receive an invitation because they were turning 17 in 2009 and at that time did not belong to the target age range of 13–16. One girl could not recall the invitation.

Twenty-two out of the 26 mothers, aged 23–66 years, who participated in the natural group discussions came to the Netherlands during the second migration wave.

### Ethical considerations

Information and informed consent letters were available in Dutch for the young women and in Dutch and Somali for the mothers. Individual participants were informed about the study during face-to-face recruitment, by telephone and e-mail, and (again) at the start of data collection. Participants were also informed about the voluntariness of the participation and that only members of the research team would have access to the interview data. We obtained informed consent and the women filled in a small demographic questionnaire.

All individual participants signed the consent letter and filled out the questionnaire. Several mothers from group discussions refused to sign and fill out the questionnaire because they were not used to giving this type of written information during their usual group gatherings. It is common that minority communities decline to provide this kind of information during research at the community level [14]. Also, participants did not feel compelled to stay for the whole session. Nevertheless, these mothers gave oral consent for participating and were actively engaged in giving information. Ethical approval is not required for this type of study in the Netherlands [23], as only particular types of behavioral research fall under the Medical Research Involving Human Subjects Act [24].

### Measures and procedures

Semi-structured interviews and natural group discussions were held. An interview guide was developed that covered topics based upon intersectionality and earlier studies that explored factors significant for the decision to participate in the prevention of cervical cancer [25, 26]. The HBM, particularly, guided the construction of probing questions.

Since there is a cultural taboo within the Somali community on openly discussing sexual matters, the sequence of the topics was carefully chosen: there were questions that participants easily could answer and then it proceeded to more sensitive topics [27]. In general, the interviews were open in nature: topics were not always addressed according to the sequence of topics presented in the interview guide, but rather to where the participants were taking the interview. However, the interviewer always made sure that all intended topics were covered.

“Natural groups” refers to groups consisting of people who know each other already from other situations, such as sports teams, work, or women’s support groups. Researching with these groups maximizes the interaction between participants, and between participants and the facilitator. It provides access to a shared group culture [14]. In this case, the women gathered regularly to discuss issues that were important to them. With the support of the chair of a Somali women’s organization and a Community Health Service (CHS) educator, natural group discussions were facilitated.

The information in the natural group discussions was collected only after JS was introduced by the moderators and she had become acquainted with the mothers who participated in the weekly group gatherings. Information on (the prevention of) HPV and cervical cancer was presented by JS to the participants at different moments. Facilitating a discussion with mothers who only recently moved to the Netherlands would have been difficult without the provision of any information on HPV and cervical cancer.

The interviews and group discussions were carried out by JS between March and June 2013. The interviews with young Somali women were conducted in Dutch, while most of the interviews with the mothers were conducted in Somali. Interviews lasted approximately 30 to 40 min. Interviewees (with the exception of one) preferred to be interviewed at a location other than home. Interviews were held at cafés, libraries, schools, and community centers. Nearly all the individual interviews were recorded and transcribed verbatim. However, five interviews with mothers were not recorded because those mothers were suspicious of recording. Short notes were taken during those interviews and immediately written out afterwards in field reports.

Two natural group discussions were held and attended by 12 and 14 mothers (aged 23–66 years). The natural group discussions were conducted in Somali, lasted approximately 20 to 40 min, and took place in community centers in two different cities. A group interview protocol was developed by JS, who moderated the focused discussions [28].

All mothers who were interviewed individually also participated in group discussions and/or the member check. Member checking is a method of validity checking, which involves taking the findings back to the participants, presenting the findings for further feedback, and ensuring that the participants agree [29, 30]. The member check, which was carried out in July 2013, was attended by 13 mothers and lasted approximately 40 min. The results were discussed to ensure that JS had comprehended the participants’ views [30]. The group discussions and the member check were recorded and transcribed verbatim.

### Data analysis

Data collection and data analysis were conducted simultaneously and iteratively. Interviews and natural group discussions were translated by JS from Somali into Dutch before analysis. The data was analyzed thematically for content and the following steps were taken: familiarizing with the data, coding the interview texts, searching for themes, reviewing and refining the themes, comparing the established themes with the entire data set, and writing the report [29, 31]. The framework of intersectionality enabled a comprehensive analysis of the themes by exploring the interactions between social factors in the women’s talk. JS coded and analyzed the transcripts: key words were assigned to pieces of text, and multiple concepts and relationships were identified. Researcher triangulation was applied by discussions in the research team on the analysis of the data. JS is a young woman who has an insider role in the Somali community. She supports the use of the HPV vaccine and Pap smears because of the proven health benefits, but also understands the concerns about unknown side effects of the vaccine in the long-term. PV, FdB, and TA are Dutch researchers with notable experience in the field of qualitative research, gender, and diversity in relation to public health. Thus, different insights were used to create and adjust codes (Table 3).

**Table 3:** The process of thematic content analysis of the interviews and natural group discussions

Phases	Themes
Phase 1	*Initial themes identified*
After six interviews: interview with three mothers and three young women.	- Perceptions of Human Papillomavirus (HPV) vaccination
	- HPV vaccination preparedness
	- Perceptions of the child vaccines
	- Perceptions of Papanicolaou Screening (Pap smears) and preparedness
	- Gender and sexuality
	- Male circumcision
	- Information on HPV vaccination and Pap smears
	- Advice for National Immunization Program (NIP) and National Screening Program (NSP)
Phase 2	*New themes identified; themes merged and renamed*
After 13 interviews: interview with seven young women.	- Perceptions of HPV vaccination and participation decision
	- Perception of other vaccination of the NIP
	- Perceptions of Pap smears and participation decision
	- Male circumcision
	- Role as parent or daughter
	- Taboo topics: Double sexual standard, Female Genital Mutilation (FGM), Sexual
	- Transmitted Infections (STIs), cancer, sex, and homosexuality
	- Information: Sharing and obtaining
	- Recommendations for NIP and NSP
Phase 3	*Intersectionality applied: final themes identified*
After 20 interviews and two group discussions:	- Barriers to participation in HPV vaccination and Pap smears: distrust towards side effects and Dutch healthcare system, lack of knowledge, language barriers, and embarrassment due to FGM and having a Dutch, male, non-Muslim practitioner
• Interview with four young women and three mothers.	
• Each group discussion with 12 to 14 mothers.	- Information and decision-making: Informal sources of information and collective decision-making
	- HPV risk perception: men’s roles in HPV transmission and cultural stigmas on HPV

To improve the credibility and transferability of this study, the RATS guidelines on qualitative research were applied^2^ [32]. Furthermore, the transparency of the study was improved by keeping a research diary and written transcripts.

## Results

In this study, we identified perceived barriers to the use of measures against cervical cancer across three major themes: (1) Somali women and preventive healthcare; (2) Language, knowledge, and negotiating decisions; and (3) Sexual standards, culture, and religion. Issues such as a lack of information and knowledge, how information is given, distrust towards the Dutch health care system and government, vaccination age, language barriers, FGM, having a Dutch, male general practitioner (GP), other traditions of information exchange, the roles of mothers, daughters, and peers in decision-making, and beliefs about sex are discussed within these three major themes, as well as how intersections of gender and culture play out in each of these themes.

### Somali women and preventive healthcare

Participants’ perceived barriers to participation include a lack of information and knowledge about the purpose of the HPV vaccination and Pap smears, about the vaccination’s possible side effects, and about how HPV is transmitted. The participants associate the HPV vaccination with unknown and negative side effects (menstrual complications, infertility and even death), and becoming an object of research:

You do not know what the future consequence of the vaccine will be and what will happen to my child. I have never received vaccination and I am completely healthy. I do not want to be a guinea pig who will undergo experiments. [N 2 (Somali mother)]

Several women mention distrust towards the ‘government’ and the Dutch healthcare system:

This [HPV vaccination] is a study. They [the government] want to know who will be the victims in the future. [N2 (Somali mother)]

Some women suspect that the government is aware of the vaccine’s side effects, yet deliberately uses it on participants to research new vaccines. The young Somali women think that negative information might be concealed by the CIb and in their interviews questioned the government’s cultural norms with regard to sex:

The government thinks: ‘you [a 13 or 14-year-old] probably had sex’, so you [a 12-year old] must take a heavy test or vaccine. I think that you [the government] encourage it [sex at a young age]. As if it is normal?! [N4 (young Somali woman)]

It becomes apparent in this interview that the girl fears that the HPV vaccination campaign normalizes sex at a young age. This is related to young Somali women’s doubts about the vaccination age. However, while they consider the age of 12 too early for girls to be confronted with sex talk, they also see the potential benefits of being prevented from acquiring HPV. The HPV vaccination is also considered as positive, as it protects women from cervical cancer and is considered as similar to other childhood vaccinations. The HPV vaccination is not seen as a measure to prevent Sexually Transmitted Infections (STIs). Aside from this, the HPV vaccine is often assumed to be mandated.

For some Somali mothers, FGM forms a barrier to getting Pap smears. Participants have stated that Somali mothers are embarrassed of what their vaginas look like. However, they stress this is not always the case, as some women consider circumcised vaginas more appealing. A Dutch, male general practitioner (GP) is considered to be a barrier to getting Pap smears among Somali mothers. However, one mother states that Dutch male GPs could not be the problem, because in Somalia women also visit male gynecologists and it is not perceived of as a problem:

Prevention is brought by Allah. You do not have to be ashamed of it [Pap smears]. (…) In Somalia, you only had male doctors. (…) We are not ashamed of our gynecologists [in Somalia]. No one is wiser than them. [Somali mother in member check]

The mothers view Somali, male practitioners as medical experts. Hence, some mothers’ barrier to getting Pap smears is most likely due to having a male practitioner with also a different cultural and religious background. They were not familiar with the possibility of having a Pap smear performed by a female practitioner.

### Language, knowledge, and negotiating decisions

Another major barrier is related to language. The Somali mothers from the second migration wave are often not fluent in Dutch, while Somali girls have access to Dutch language and culture through school. Hence, some young Somali women have to translate information about the HPV vaccination to their mothers, which daughters then sometimes perceive as a barrier. As a consequence, they have not always informed their mothers.

Participants explain that the formal leaflet is read with suspicion, or not read at all. Some mothers perceive information letters as not relevant to them, and express the need to receive oral information from a known person. A major source of information on the prevention of cervical cancer is cultural peers:

We do not understand the situation of this country. We do not understand what is written in Dutch in the letter. So you ask other people: ‘Did you get the letter on the vaccination of your child?’ (…) How you will understand the information depends on the person who explains it. [A mother in group discussion]

Furthermore, daughters and mothers discuss and exchange information on the HPV vaccination with each other, whereby the mothers’ opinions on the HPV vaccination are often decisive. Daughters tend to accept the views of their mother and their peers, and mothers tend to accept the views of their Somali peers.

Freedom in making an individual decision with regard to HPV vaccination is preferred by the Somali girls because it concerns their health. Mothers are less content with the idea of individual decision making because it restricts their ability to determine their daughters’ health choices. They also perceive their daughters as influenced by the ‘dominant’ Dutch culture, in which decision making is individualized, and to which they think they should adapt:

Every mother tells a lie to herself [and thinks]: ‘My daughter is great.’ While the daughter was just two months old when she arrived in the Netherlands, she is now 22 or 24 years old. What is the outcome? The child takes over the [Dutch] culture. Your child will not take over your [Somali] culture. Who is stronger than you? The people your child associates with. These people give her freedom. So we are obligated to vaccinate or examine her. [A mother in group discussion]

The mothers’ lack of knowledge and lack of Dutch language skills largely influences their roles in the decision-making process, and this seems more influential than the dissatisfaction with or suspicion towards Dutch culture and healthcare.

### Sexual standards, culture, and religion

Susceptibility to HPV is perceived to be low for Somali girls because they are expected to not engage in premarital sex. The sexual behavior of Somali women is seen as different from European, Dutch women. Being a virgin before marriage and being obedient to your faith are mentioned as important religious values. Dutch culture is criticized because both girls and boys are free to have sex, whereas in Somali culture only boys are allowed to have premarital sex. This double sexual standard does not correspond with most Somali peoples’ religion, which forbids premarital sex for both boys and girls. The young women and mothers accept the presence of a double sexual standard. However, the young women are also critical:

I think it is stupid [the double sexual standard]. I think you should teach a girl how she could stand up for herself. Boys should learn more rules [and] be taught discipline, so that there is a balance [between boys and girls]. [N 14 (young Somali woman)]

Somali women who are sexually active before marriage are stigmatized as sexually promiscuous and experience ‘eternal shame’ in the Somali community:

The worst thing that can happen is that he makes a girl pregnant, which of course is awful, but the boy can walk away. If she is sexually active and gets pregnant, then it is a problem. In Islam, you cannot have an abortion. So you have to take care of the child and you [will] have an eternal shame. Even if she was with a boy who she would marry, everyone would [still] think: ‘She slept with every guy, because she has a child.’ [N 11 (young Somali woman)]

This idea of eternal shame instills fear in young Somali women and mothers. The mothers use social control and education on Islamic norms as ways to promote chastity among daughters and to prevent stigma. With the cultural double sexual standard on the virginity of girls, the HPV vaccination is not considered necessary. Yet, some girls criticize it by expressing their discontent with the Somali cultural norm that stigmatizes girls who are sexually active before marriage.

Most participants also have estimated the risk of HPV as low because they had not been aware that men can be infected with HPV and transmit the virus to women. Only one mother has mentioned the causal relationship between male circumcision and a lower risk of cervical cancer, which may explain her perceiving Somali people’s susceptibility to HPV as low. After receiving information, participants often have stated that boys should be vaccinated against HPV or that men should be tested for it before marriage to protect girls and women:

I think it is scary, because what if [you get HPV]? I find it strange: why do not they [the government] have vaccines for boys? Or is that not possible? [N 14 (young Somali woman)]

However, some participants anticipate difficulties with testing and vaccinating Somali men for HPV. Participants have mentioned that Somali men may assume that their partners do not trust them and feel offended.

Although most participants believe they can control the risk of cervical cancer through sexual behavior, the Somali mothers particularly believe that all diseases are determined by God and indicate low self-efficacy. However, there is also individual religious responsibility to improve one’s health and prevent disease.

When informed by JS that cervical cancer is a common cancer in women in Somalia, the perceived severity of HPV infection and cervical cancer seemed to increase. It has been mentioned that cancer is a sensitive discussion topic within the Somali community. This is also the case for homosexuality, which could explain why most participants did not know about homosexual transmission of HPV. The stigma and discrimination of homosexuality within the Somali community is recognized:

I personally think that they [homosexual people] should do whatever they want. If I think of the Moroccan youth, all so tough [macho]…He [a Moroccan person] really does not choose to be gay if he will be disowned, scolded [and] beaten up. I do not think that someone would choose for that. He also wants to have kids and a wife, right? Look, maybe Dutch boys like it [being homosexual], but I really do not think that certain people choose for it. [N 18 (Somali girl)]

Homosexuality is seen as a lifestyle choice for Dutch people, related to the sexual freedom within Dutch culture. The women and girls do not mention the possible transmission of HPV between women.

## Discussion

This study explores the perceptions of Somali women in the Netherlands about the prevention of cervical cancer. Based on the HBM and intersectionality, the girls’ and the mothers’ perceptions have been distinguished in the following themes, in which gender and culture play particular roles: (1) Somali women and preventive healthcare; (2) Knowledge, language, and negotiating decisions; and (3) Sexual standards, culture, and religion.

With regard to the HBM, Somali women perceive many barriers to the use of preventive cervical cancer care, such as a lack of information and knowledge, distrust towards the HPV vaccination and side effects, and distrust towards the ‘government’ and the Dutch healthcare system, as reported earlier [14, 25, 33]. Especially Somali mothers from both migration waves express distrust towards the Dutch health care system, which may be explained by war trauma and FGM experienced in the country of origin (Table 1). Also, given the Somali cultural context, young women consider 12-year old girls as too young to discuss sex. However, they perceive potential benefits from HPV prevention: it protects women from cervical cancer and, just as childhood vaccinations, it is assumed to be mandated and not linked to STIs. Pap smears performed by a male practitioner and FGM have been reported earlier as barriers to participation [13, 16, 34]. Somali mothers are said to be reluctant to visit a Dutch, male practitioner, which seems related to Dutch doctors’ unfamiliarity with aspects of their cultural and religious background, such as FGM, the value of chastity, their mother tongue, and ways of communicating their illnesses [14], rather than because of their gender [13].

In the second theme, about how decisions in regards to the HPV vaccination are negotiated between mothers and daughters, we see that Somali mothers’ poor Dutch language skills - particularly from the second migration wave - give their children the role of translating information about HPV vaccination. This, in turn, is perceived as a barrier to participation by Somali girls. Furthermore, these Somali women live by certain traditions of information exchange. The formal leaflet is read with suspicion, or not read at all. In the Somali, female community, cultural peers are a major source of information, and decision-making on the medical prevention of cervical cancer takes places within social relations. Mothers and daughters exchange information on the HPV vaccination with each other, even though the mothers’ opinion often forms a cue to (reject) action. Some mothers perceive a limit of freedom for determining their daughters’ health actions because of the experienced information gap and the ‘dominant’ Dutch culture, in which decision-making is individualized.

The participation of Somali women in measures to prevent cervical cancer is heavily influenced by cultural beliefs and behaviors. An intersectional approach allows us to thoroughly explore how social identities relate to each other and how the intersection of these identities influences the participation of Somali women in measures to prevent cervical cancer. In the third theme, on sexual standards, and in contrast with Dutch mothers and girls, the perceived susceptibility to HPV is lower among Somali women, because they are expected to abide by the religious and cultural norm of virginity before marriage. In addition, in the Somali community, a cultural double sexual standard is present that allows only boys to have premarital sex. Although most girls in the study accept this double sexual standard, some express criticism towards it. Nevertheless, girls who have premarital sex are stigmatized in the Somali community and, thus, mothers often promote chastity among their daughters, as mentioned earlier [35]. Most of the girls have internalized the traditional value of chastity and express the importance of virginity, independent of educational background. From a Western point of view, chastity may have a negative impact on a girl’s self-image in regard to sexuality and limit her ability to discuss sexual matters with her future partner(s) [35]. However, from a religious Islamic and cultural perspective, the value of chastity may enhance a girl’s self-image concerning sexuality in the sense that virginity before marriage is believed to protect the body from sexual hazards such as cervical cancer, STIs, and teenage pregnancy. Chastity can be an expression of self-respect or respect for one’s own body [36].

The Somali women in the study had not been aware of possible susceptibility to HPV through partners, even though they had been aware of a cultural double sexual standard, as also found in a study among Turkish and Moroccan mothers [33]. Also, cancer is perceived as a sensitive topic by the women [14]. The Somali mothers particularly attribute cervical cancer to metaphysical beliefs such as fate and God’s will [37]. However, the Islamic faith also plays the role of a cue to action: some participants state that their religion supports preventive care and/or medicine to improve health.

Combining an intersectional approach with the Health Belief Model provides contextualized knowledge on how the women’s different positions in aspects such as age, migration status and language skills, religion, or culture are interdependent and influence individual perceptions of preventive measures and health. According to Hankivsky et al. [19], intersectionality directs attention to health issues that are less well understood for certain groups, in our case, Somali women and the prevention of cervical cancer. Gendered, cultural, and social structures produce and reproduce inequalities that intersect, overlap, and reinforce each other in shaping a person’s health status. To prevent disease, it is important to gain an understanding of how women’s perceptions are contextualized, and we must also target our interventions towards institutions, such as healthcare and health promotion programs, rather than target individuals.

### Strengths and limitations to the study

This study has some limitations. First, discussion within natural existing groups may have inhibited truthful disclosure. However, the women in this study generally have felt safe to discuss sensitive topics within their already established group of cultural peers. Besides, according to the organizations who helped recruit interviewees, the women would not have accepted a structured focus group.

Second, in a few cases, a participant’s friend was present during an interview and some interviews were held in public spaces, which may have influenced the responses to some questions. Third, group discussions and interviews with Somali mothers were conducted in Somali. Meanings of some responses by participants may have inadvertently changed during translation, which could affect the validity of this study [38]. Fourth, some mothers have declined audio-recording, and nuances may have been lost because the information gathered from them is based on extended field reports.

Finally, although the level of education of Somali people in the Netherlands is generally low (Table 1), most young Somali women in this study followed higher education. The researcher has had limited access to lower educated young Somali women, which could be attributed to her own educational background and the short time available for the research. It is difficult to say how such selection bias influences the results. In hindsight, the results do not show major differences in perceptions towards prevention of cervical cancer between higher educated and lower educated girls. Most girls in this study have expressed the importance of these traditional values, independent of their educational background. The recruitment of girls with lower education levels could perhaps have led to more diverse results and saturation [29]. More studies including quantitative research could be used to increase the validity of our findings.

This study has several strengths. First, the recruitment methods applied in this study led to low-cost recruitment and the inclusion of a large number of Somali people. JS’s Somali background has created easy access to the community [39]. Second, this also has helped the participants feel more comfortable and disclose information, enhancing the ecological validity of the study. However, having the same background as the participants also could become a barrier: JS has a reputation to maintain within the community. Possibly, this made it difficult for her to pose questions about sensitive issues, which a Dutch researcher would not have experienced.

Third, data triangulation has been reached by collecting data from different sources, including individual interviews and natural group interviews. Fourth, researcher triangulation and a member check have been applied to increase the validity of the study. During the member check, the researcher established credibility by presenting all findings derived from the interviews and group discussions. These findings were recognized, validated, and further clarified by the participants. Finally, some mothers have been recruited from the first, and other mothers were recruited from the second migration wave. Mothers who are only recently in the Netherlands may be less knowledgeable about the Dutch health system and its preventive measures.

We identified several implications of our study. More information that clarifies misperceptions around the prevention of cervical cancer and the Dutch health care system is important. Furthermore, our study shows how a reversal of parent-child roles can take place as a consequence of parents’ difficulties in navigating the new country in which they live. This study also shows that religion is subordinate to culturally defined gender norms that control the behavior of boys and girls by stigmatizing girls having premarital sex. Moreover, this study shows that Somali women in the Netherlands are often not aware of men’s roles in HPV transmission. Despite the finding that men can be infected with HPV and transmit the virus to women, there are no nationally funded HPV vaccination programs or HPV DNA testing programs targeting men in Europe (with the exception of Austria). Thus, in order to protect the sexual health of ethnic minorities in the Netherlands, it is important to not only consider informing ethnic minorities about men’s roles in HPV transmission, but also to implement nationally funded HPV-related preventive care for men.

This study also shows how social factors influence Somali women’s health beliefs, which underlie participation in cervical cancer prevention. One can question whether the current cervical cancer prevention programs, the ways they are promoted, and by whom, fits with Somali culture. The framing of HPV vaccination messages as specifically preventing cervical cancer affecting Somali women in Somalia and in the Netherlands may increase the perceived severity of HPV and thus increase acceptability of vaccination amongst the Dutch-Somali population [40]. Also, the message that male circumcision has a protective effect against cervical cancer must be presented with caution to Somali women.

Oral education in Somali may reach mothers best [14, 41], as Somalia is known for its long tradition of poetry [41]. Hence, culturally sensitive information on cervical cancer prevention and the Dutch healthcare system can be provided through poetry or theater, and developed together with the target group. In addition, group meetings in which mothers can ask questions, reflect on cultural values, and exchange experiences with each other is recommended. Also, the use of cultural brokers in health care settings, who can identify with and have knowledge of a client’s cultural background as well as the Dutch health care system, might increase the demand for preventive health care [39].

Teachers can provide and discuss culturally appropriate information targeting the double sexual standard in schools. It is also recommended to take the cultural stigma on sexual matters into account and extend the HPV vaccination to an older age, as suggested earlier [33].

This study has unexpectedly increased Somali women’s awareness of their own cultural values and norms, and has stimulated them to reflect on perceptions different from their own. For some, it has even empowered them to change their attitudes towards the prevention of cervical cancer [28]. Forms of participatory research therefore seem appropriate for studying taboo topics, most likely for other marginalized groups as well.

## Conclusions

To our knowledge, this study is one of the few studies in Europe that explores the perceptions of Somali women regarding the prevention of cervical cancer. This study contributes to the literature by demonstrating that Somali women face multiple obstacles, including language barriers, a lack of knowledge about the existence of certain health services, and distrust of side-effects and the Dutch health care system, which limit their access to health care.

More research is recommended to explore migrant groups’ distrust towards Dutch health care, how to establish trusting relationships, the levels of awareness among ethnic minorities about men’s role in HPV transmission, the possible negative effects of a double sexual standard on susceptibility to HPV, and whether such knowledge plays a role in the consideration to take preventive cervical cancer measures or not. More studies are needed on the diversity in perceptions of the prevention of cervical cancer within and between ethnic minorities in order to promote equal access to health care for everyone. An intersectional framework, grounded in the HBM, is recommended for future studies.

## Footnotes

1 This poem has been written for this study and is intended for distribution.

2 The RATS guidelines on qualitative research are reporting guidelines provided for qualitative research reviews. They review the following four aspects of a manuscript: Relevance of study question, Appropriateness of qualitative method, Transparency of procedures, and Soundness of interpretive approach (RATS).
